# Immobilization of Lipase from *Penicillium* sp. Section *Gracilenta* (CBMAI 1583) on Different Hydrophobic Supports: Modulation of Functional Properties

**DOI:** 10.3390/molecules22020339

**Published:** 2017-02-22

**Authors:** Daniela F. M. Turati, Wilson G. Morais Júnior, César R. F. Terrasan, Sonia Moreno-Perez, Benevides C. Pessela, Gloria Fernandez-Lorente, Jose M. Guisan, Eleonora C. Carmona

**Affiliations:** 1 Department of Biochemistry and Microbiology, Biosciences Institute, Universidade Estadual Paulista (UNESP), 13506-900 Rio Claro, SP, Brazil; Daniturati@hotmail.com (D.F.M.T.); ecarmona@rc.unesp.br (E.C.C.); 2 Instituto de Investigación en Ciencias de la Alimentación (CIAL), CSIC-UAM, 28049 Madrid, Spain; wjunioreq@gmail.com (W.G.M.J.); b.pessela@csic.es (B.C.P.); g.f.lorente@csic.es (G.F.-L.); 3 Instituto de Catálisis y Petroleoquímica (ICP), CSIC-UAM, 28049 Madrid, Spain; 4 Pharmacy and Biotechnology Department, School of Biomedical Sciences, Universidad Europea, 28670 Madrid, Spain; chaspy_27@hotmail.com

**Keywords:** enzyme immobilization, enzyme stabilization, fish oil hydrolysis, fish oil ethanolysis, Omega-3 production

## Abstract

Lipases are promising enzymes that catalyze the hydrolysis of triacylglycerol ester bonds at the oil/water interface. Apart from allowing biocatalyst reuse, immobilization can also affect enzyme structure consequently influencing its activity, selectivity, and stability. The lipase from *Penicillium* sp. section *Gracilenta* (CBMAI 1583) was successfully immobilized on supports bearing butyl, phenyl, octyl, octadecyl, and divinylbenzyl hydrophobic moieties wherein lipases were adsorbed through the highly hydrophobic opened active site. The highest activity in aqueous medium was observed for the enzyme adsorbed on octyl support, with a 150% hyperactivation regarding the soluble enzyme activity, and the highest adsorption strength was verified with the most hydrophobic support (octadecyl Sepabeads), requiring 5% Triton X-100 to desorb the enzyme from the support. Most of the derivatives presented improved properties such as higher stability to pH, temperature, and organic solvents than the covalently immobilized CNBr derivative (prepared under very mild experimental conditions and thus a reference mimicking free-enzyme behavior). A 30.8- and 46.3-fold thermostabilization was achieved in aqueous medium, respectively, by the octyl Sepharose and Toyopearl butyl derivatives at 60 °C, in relation to the CNBr derivative. The octyl- and phenyl-agarose derivatives retained 50% activity after four and seven cycles of *p*-nitrophenyl palmitate hydrolysis, respectively. Different derivatives exhibited different properties regarding their properties for fish oil hydrolysis in aqueous medium and ethanolysis in anhydrous medium. The most active derivative in ethanolysis of fish oil was the enzyme adsorbed on a surface covered by divinylbenzyl moieties and it was 50-fold more active than the enzyme adsorbed on octadecyl support. Despite having identical mechanisms of immobilization, different hydrophobic supports seem to promote different shapes of the adsorbed open active site of the lipase and hence different functional properties.

## 1. Introduction

Lipases (triacylglycerol acyl hydrolase, EC 3.1.1.3) constitute a class of enzymes that catalyze the hydrolysis of ester bonds from long chain triacylglycerol with low solubility in water, giving them the property to catalyze at an oil/water interface [1]. In addition to hydrolysis, these enzymes are capable of catalyzing the reverse reaction in organic media, esterifying fatty acids and glycerol into triglycerides, as well as acting on transesterification and alcoholysis reactions, among others. Microbial lipases therefore emerge in the current industrial scenario due to their wide range of applications, which may be directly related to their diverse functions [2,3].

Enzyme immobilization is a primordial requirement to overcome common bottlenecks which hinder the large-scale application of biocatalysts at industrial level. This way, the immobilization technique/protocol should be designed in such a way to improve enzyme properties, in relation to activity, selectivity, performance in organic solvents, pH tolerance, heat stability, or the functional stability [4,5,6].

Immobilization of lipases on hydrophobic supports is based on a peculiar mechanism, the so-called interfacial activation [7,8]. It is well recognized that lipases have a closed structural conformation wherein the active center is isolated from the reaction medium by a polypeptide chain named lid or flat, which possess a hydrophobic internal face interacting with hydrophobic surroundings of the active center. In the presence of a hydrophobic surface, the lid changes its position exposing the hydrophobic pocket to the medium, easing substrate access to the active site. This open form is quite unstable in aqueous and homogenous medium, but becomes stabilized by adsorption on hydrophobic surfaces, which can be originated from a substrate or hydrophobic supports. Adsorption on hydrophobic supports has been widely used for lipase immobilization, since the technique is rapid, simple, and of low cost and high selectivity, allowing at one time purification, immobilization, hyperactivation, and stabilization [7,8,9,10,11,12,13,14,15,16]. A limitation of this type of biocatalyst, however, is the enzyme release from the support which can occur in the presence of some reaction products, an important factor that should be taken in consideration during the selection of an industrial lipase biocatalyst [17].

A key application of immobilized lipases is for the production of polyunsaturated fatty acids (PUFA), such as Omega-3, important in the food industry. This compound is found mostly in marine-derived products and can be obtained by hydrolysis or alcoholysis of fish oils catalyzed by lipases. Omega-3 is a nutrient that promotes positive effects in human health, constituting an increasing market of dietary supplements and nutraceuticals that contain these PUFA. For young people, they are required at high levels by the brain and retina, improving learning ability, mental development, and visual acuity, while in adults it is considered beneficial for prevention of cardiovascular diseases and by improving muscle function in older women [18,19].

Considering the current global scenario of the search for healthier dietary supplements, the main objective of this work was to immobilize the lipase produced by *Penicillium* sp. section *Gracilenta* (CBMAI 1583). The fungal strain was isolated from soil at the Atlantic Rainforest region (São Paulo State, Brazil) [20], and produces a lipase with 52.9 kDa (estimated by denaturing electrophoresis), which is optimally active at pH 4.0 and 70 °C (unpublished data). The immobilization was evaluated using hydrophobic supports—i.e., agarose based butyl-(But), phenyl-(Phe) and octyl-Sepharose (Oct), acrylic Toyopearl (Toyo), and macroporous Lewatit VP OC 1600 (Lew) and octadecyl Sepabeads (Sep)—in order to obtain highly active and stable biocatalysts. The properties of the immobilized enzyme were compared to the cyanogen bromide derivative, which emulates the properties of the soluble enzyme but without problems caused by molecules interaction. The derivatives were characterized and applied in reactions to obtain Omega-3 fatty acids and ethyl esters from fish oil, a very appreciated product in food and pharmaceutical industries.

## 2. Results and Discussion

### 2.1. Immobilization on Hydrophobic and Cyanogen Bromide Supports

Immobilization of lipases occurs via a peculiar mechanism known as interfacial activation [7,8], This naturally-occurring mechanism was used as a tool to immobilize several microbial lipases in many different hydrophobic supports proving to be a simple and efficient method that can allow selective purification, due to lipase adsorption even at low ionic strength, giving rise to purified-stabilized derivatives [9,10,11,12,13,14,15,16].

The *Penicillium* sp. (CBMAI 1583) lipase was hydrophobically immobilized by performing incubation during 90 min for But, Phe, and Oct and 120 min for Toyo, Lew, and Sep supports. All immobilization processes presented high yield (above 75%) and the expressed activity ranged from 54.2% to 144.9% (Table 1).

Immobilization on octyl Sepharose support presented 71.4% yield and the immobilized enzyme was 1.5-fold activated producing a derivative with 20.9 U/mg support. In addition to purification and stabilization, the fixation of the opened enzyme conformation facilitates access of the substrate to the active site, commonly resulting in hyperactivated lipase biocatalysts [7,8].

Fernández-Lorente et al. [12] compared immobilization of commercial CALB (*Candida antarctica* lipase B), TLL (*Thermomyces lanuginosus* lipase), BTL (*Bacillus thermocatenulatus* lipase), and Lecitase Ultra on both agarose-based and Toyopearl supports, and the expressed activity ranged from 50% with Lecitase adsorbed on butyl Toyopearl to a seven-fold hyperactivation with TLL adsorbed on octyl agarose. Similar to *Penicillium* sp. (CBMAI 1583) lipase derivatives, these authors verified the octyl agarose rendered derivatives expressing the highest activities for all commercial enzymes. These results might be related to the close contact between the enzyme active center and the support; thus, changes in the internal morphology or in the groups coating the support may greatly alter enzyme properties. Agarose is a strongly hydrophilic, lyophilic and inert colloid that can reversibly form stable and firm gels, and its suitability for immobilization is confirmed by the ability to form derivatives [21]. The evaluated agarose-based supports are activated with different hydrophobic groups and highly cross-linked structures, offering an almost planar surface for enzyme interaction. Toyopearl, an acrylic support formed by thin crossed fibers that might be even smaller than the lipase molecules, also possesses certain hydrophobicity. Lewatit and Sepabeads are macroporous cross-linked methacrylate polymers, which might allocate lipases inside their structure [12,16]. Moreover, coating the fiber surfaces with butyl, phenyl or octyl groups may reinforce the hydrophobic character of each matrix. Therefore, the interaction of a lipase with different supports may result in variable immobilization and importantly produce catalysts exhibiting different characteristics.

CNBr support immobilizes enzymes by one very stable bond through the terminal amine group, which has pK around 7–8, allowing immobilization under very mild conditions [22]. Consequently, by avoiding multipoint covalent attachments, this derivative mimics soluble enzyme behavior and constitutes a good model to study enzyme properties in absence of intermolecular phenomena [23]. Due to the use of mild conditions, immobilization yield was low (21.0%) and the CNBr derivative presented 1.4 U per milligram of support.

The hydrophobic derivatives were incubated in increasing Triton X-100 concentration and the amount necessary to completely release the lipase was a measure of the adsorption strength (not shown). Sep required the highest detergent amount to complete enzyme desorption (5.0%). This fact may be due to morphology and hydrophobicity, since it is a macroporous acrylic support that can allocate lipase molecules inside its structure and it is functionalized with an 18-carbon alkyl chain, therefore being the most hydrophobic support and the one wherein lipase adsorption strength was higher. Lew derivative, likewise a macroporous support functionalized with octyl groups, required intermediate Triton amount (1.0%), while But, Oct, Phe, and Toyo required lower amounts (0.15%–0.20% Triton) to total enzyme desorption from the support. The lower Triton amount required for desorption may be related to unfavorable geometrical congruence between the lid and the active center of the enzyme interacting with hydrophobic groups of the supports [12]. Fernández-Lorente et al. [12] observed that higher detergent amounts are necessary to release the lipase from *Bacillus thermocatenulatus*, *Thermomyces lanuginosus*, and Lecitase immobilized on Toyo, suggesting that adsorption strength is determined not only by support structure and hydrophobicity, but also by enzyme characteristics, such as size of the lid and number of hydrophobic residues.

### 2.2. Derivatives Characterization

#### Effect of pH and Temperature on Lipases Activity

**Figure 1:**
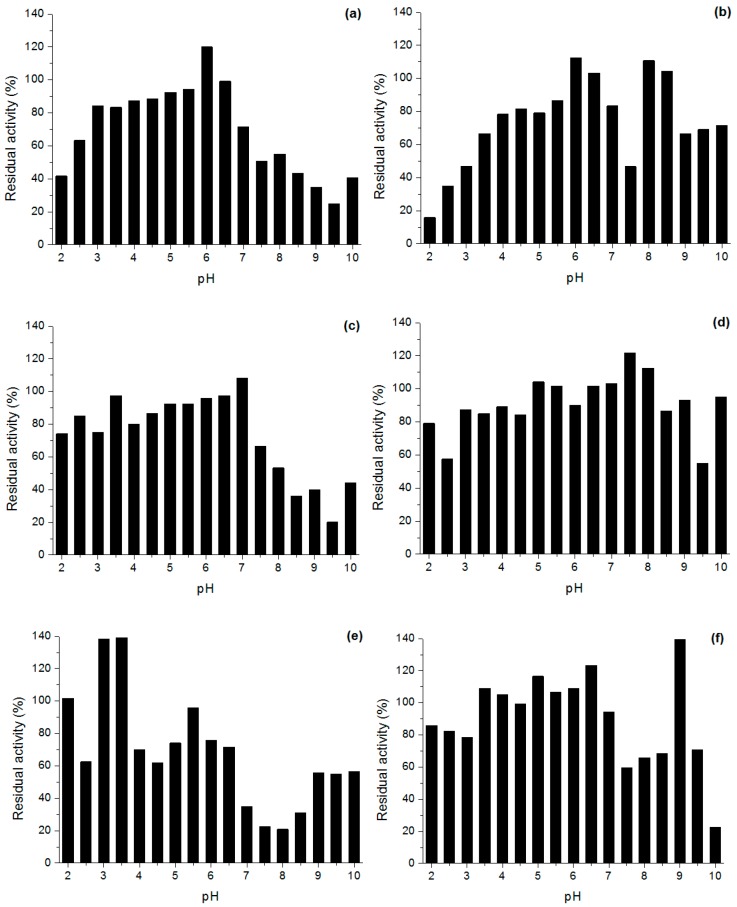

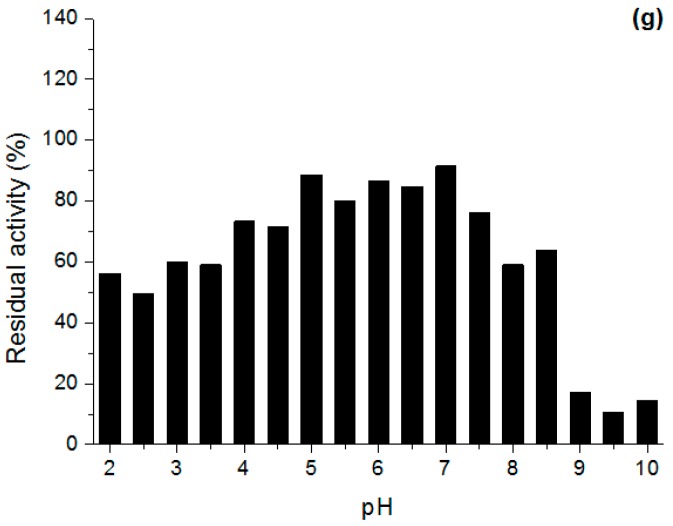
Stability in different pH of the *Penicillium* sp. (CBMAI 1583) lipase derivatives in aqueous media. (**a**) Butyl Sepharose; (**b**) phenyl Sepharose; (**c**) octyl Sepharose; (**d**) Toyopearl butyl 650 M; (**e**) Lewatit VP OC 1600; (**f**) octadecyl Sepabeads and (**g**) CNBr derivatives. The derivatives were incubated at 1:10 (*w*/*v*) proportion in the following buffers: 0.5 M glycine-HCl pH 2.0 and 2.5; McIlvaine pH 3.0–8.0; 0.5 M Tris-HCl pH 8.5 and 9.0; and 0.5 M glycine-NaOH pH 9.5 and 10.0; for 24 h at 25 °C.

When incubated at different pHs, the derivatives in general presented higher stability in acid-neutral pH and lower activity in more alkaline pH (Figure 1).

In general, all derivatives were more stable than CNBr derivative, but different stabilization was verified among the diverse derivatives in different pH. Oct and mainly Sep stabilized the enzyme in a wider pH range and these derivatives retained more than 50% of activity in the pH range from 2.0 to 9.5, while But and Oct derivatives stabilized the enzyme in more acid pH. Lew and Sep presented the worst results in terms of pH stabilization.

**Figure 2:**
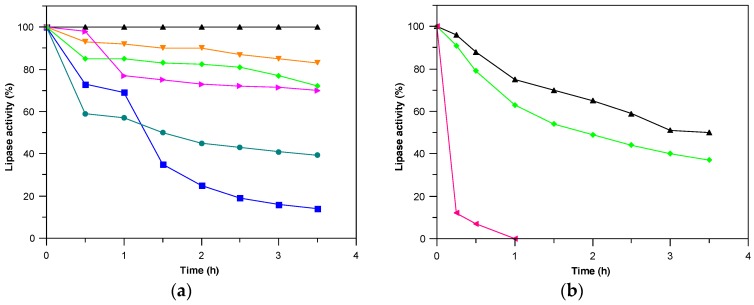
Thermal inactivation of the *Penicillium* sp. (CBMAI1583) lipase derivatives. Incubation was carried out in 5 mM sodium phosphate buffer pH 7.0 at (**a**) 50 and (**b**) 60 °C. Lipase activity (%) of (▲) Toyopearl butyl 650 M; (■) Lewatit VP OC 1600; (●) octadecyl Sepabeads; (▼) butyl Sepharose; (►) phenyl Sepharose; (♦) octyl Sepharose and (◄) CNBr derivatives.

In order to evaluate and compare thermal stability, *Penicillium* sp. (CBMAI 1583) lipase derivatives were incubated in pH 7.0 at different temperatures (Figure 2). At 50 °C, But, Phe, Oct, and Toyo derivatives were quite stable, retaining more than 70% of activity after 3.5 h-incubation, while Lew and Sep derivatives were barely stable (Figure 2a). At 60 °C, Toyo and Oct were very stable, being 46.3- and 30.8-fold more stable than CNBr derivative, respectively (Figure 2b).

Data presenting deactivation constant and half-lives of the different derivatives at different temperatures are summarized in Table 2. Due to the high stabilization, adsorption on hydrophobic supports showed to be a successful alternative for immobilization of the *Penicillium* sp. (CBMAI 1583) lipase. These results are better than others previously reporting two-fold stabilization for the TLL immobilized on poly-hydroxybutyrate particles and CRL (*Candida rugosa* lipase) immobilized on Sepabeads [24,25].

In addition to hydrolysis of long chain triacylglycerol, lipases are capable of catalyzing the reverse reaction in organic media, esterifying fatty acids and glycerol into triglycerides, as well as acting on other reactions, such as transesterification and alcoholysis [2,3]. In this sense, lipases are of great interest in organic chemistry, and evaluating their stability in organic media is essential for further reaction design.

The Oct derivative was incubated for 2 h with glycerol, DMSO, acetonitrile, ethanol, tert-amyl alcohol, and cyclohexane. In Table 3, the results are displayed and solvents are organized according to their hydrophobicity, meaning that the higher the partition coefficient logarithm (Log *P*) the more hydrophobic the solvent [26].

The Oct derivative was very stable in glycerol, retaining more than 93% of activity after incubation. The other solvents negatively affected activity of the immobilized enzyme—i.e., intermediate activity was verified with DMSO (dimethyl sulfoxide) and cyclohexane—and no activity was detected after incubation with acetonitrile, ethanol, and tert-amyl alcohol.

In general, polar miscible water solvents are more destabilizing than hydrophobic immiscible solvents, but there is no consensus in relation to lipase stability in the presence of different solvents. It is suggested that non-polar solvents may promote changes in the equilibrium between the open and closed conformations of lipases and also modify substrates and products solubility; while polar solvents may remove the water solvation shell [27]. In this study, no correlation between Log *P* and stability was observed; with stability being more closely related to solvent chemical nature than to its hydrophobic properties.

### 2.3. Hydrolysis of Fish Oil in Aqueous Media

But, Phe, and Oct derivatives were applied to the production of eicosapentaenoic acid (EPA) and docosahexaenoic acid (DHA) Omega-3 fatty acids from sardine oil hydrolysis (Table 4, Figure S1) using a biphasic water-immiscible co-solvent (cyclohexane) system to allow oil manipulation, especially at low concentrations [14]. In this case, the lipase inside a porous support can only hydrolyze oil molecules partitioned in the aqueous phase of the system.

Phenyl was the most active derivative, releasing 1.2-fold more PUFA than the free enzyme, whereas but derivative was the less active, producing 3-fold less PUFA than that verified with Phe. Intermediate PUFA amounts were observed with CNBr and Oct derivatives. In these kinds of support, lipases cannot undergo interfacial activation by oil drops, being able to hydrolyze only oil molecules partitioned into the aqueous phase of the biphasic reaction system [13]. The results are comparable to those reported for fish oil hydrolysis under similar conditions by many lipases adsorbed on hydrophobic supports. Fernández-Lorente et al. [13] applied the lipases from *Thermomyces lanuginosus* immobilized on octyl Sepharose presenting initial activity of 0.06 μmol of PUFA per minute per g of derivative, lower than that found in this study (0.073 μmol of PUFA per min per g of derivative). Fish oil hydrolysis by *Hypocrea pseudokoningii* lipase immobilized on butyl Sepharose [28] shows initial activity of 0.03 μmol of PUFA per min per g of derivative, similar to that obtained in the hydrolysis of fish oil by the lipase from *Penicillium* sp. (CBMAI 1583) immobilized on the same support.

In relation to selectivity, all derivatives hydrolyzed EPA at a higher rate than DHA (selectivity > 1). Among the hydrophobic derivatives, phenyl was the most selective, while much higher selectivity was observed with CNBr derivative. Similar to our results, Pereira et al. [28] applied the lipase from *Hypocrea pseudokoningii* immobilized on CNBr and glyoxyl-agarose to hydrolyze sardine oil, which was performed in a water-organic solvent two-phase system at pH 6.0 and 25 °C, obtaining selectivity of 3.0 for CNBr derivative and 7.0 for glyoxyl-agarose derivative. Morais Júnior et al. [29] used the immobilized lipase from *Candida rugosa* for sardine oil enrichment with Omega-3, obtaining the values 5.0 and 4.0 for selectivity, with the octyl Sepharose and CNBr derivatives, respectively. Fernández-Lorente et al. [13] studied the hydrolysis of fish oil at pH 7.0 and 25 °C, using the lipase from *Candida antarctica* B immobilized on porous supports, and achieved 1.5 selectivity. Consistently with these studies, this may be explained by steric hindrance of the DHA molecule produced by multiple folds in *cis*-type unsaturation along its hydrocarbon chain. In addition, DHA is commonly esterified in position *sn*-2 of triacylglycerol, hampering lipase access to the ester bond [30]. Thus, hydrophobic derivatives of *Penicillium* sp. (CBMAI 1583) lipase can be very useful to obtain mixtures of EPA and DHA from sardine oil, while the CNBr derivative could be used to enrich EPA content in the fish oil.

### 2.4. Ethanolysis of Fish Oil in Organic Media

Ethanolysis converts triacylglycerol into ethyl esters and glycerol, in organic medium with ethanol. Ethanolysis of fish oil promotes the synthesis of ethyl esters containing Omega-3 fatty acids, which are of interest within the food industry. Enzymatic synthesis of ethyl esters is attractive compared to chemical reactions since it can be executed under mild conditions avoiding the formation of undesirable byproducts [31].

In this work, ethanolysis of sardine oil was performed with the derivatives Sep, Lew, and Toyo from *Penicillium* sp. (CBMAI 1583) lipase (Table 5). Due to their acrylic structure, they can be dried since, contrarily, an increase in water content would favor hydrolysis over synthesis. Anhydrous medium was maintained by using dried derivatives and also by adding molecular sieves to the reactor. Organic solvents may be advantageous by facilitating handling of oils and by modulating and improving the activity-selectivity of immobilized enzymes.

Lew was the most active derivative in all evaluated conditions. The activity observed with this derivative in cyclohexane was similar to that verified in the control and discrimination between EE-EPA and EE-DHA was slightly higher in the latter. When the reaction was performed with tert-amyl alcohol the activity was increased by more than five-fold. Contrarily, Sep and Toyo were much more active in absence of cyclohexane or tert-amyl alcohol. In fact, Toyo showed no activity in the presence of cyclohexane. In relation to selectivity, Sep was more selective without cyclohexane or tert-amyl alcohol while Toyo in the reaction with tert-amyl alcohol produced only EE-EPA.

Moreno-Pérez et al. [31] performed selective fish oil ethanolysis with CALB, TLL, and RML adsorbed on octadecyl Sepabeads in medium with cyclohexane or tert-amyl alcohol. The activities were higher (0.0736 μmol of EE-PUFA per min per mg of immobilized lipase for CALB with cyclohexane) probably due to the higher protein amount loaded in these derivatives. In terms of selectivity, discrimination between EE-EPA and EE-DHA varied from 1 to 29. These results confirm that selective ethanolysis depends on the type of enzyme, support, and solvent. In this case, ethanolysis performed with Lew in tert-amyl alcohol could be useful for ethyl esters synthesis and EE-EPA could be purely obtained by ethanolysis with Lew in tert-amyl alcohol. Cipolatti et al. [32] applied the lipase from *Thermomyces lanuginosus* immobilized on support synthetized with polyurethane and polyethylene glycol to produce PUFAs, which was performed at 37 °C, obtaining a selectivity of 12.4.

### 2.5. Derivative Reuse

**Figure 3:**
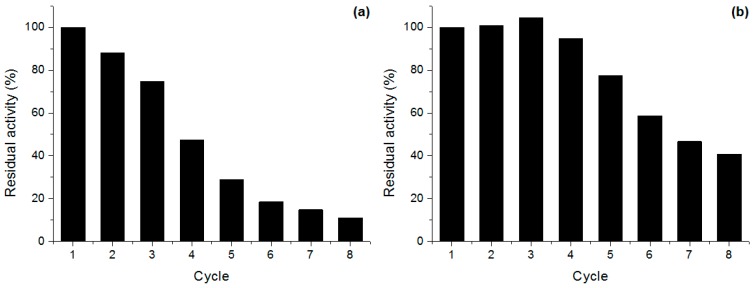
Reuse of (**a**) octyl and (**b**) phenyl derivatives of *Penicillium* sp. (CBMAI 1583) lipase on *p*NPP hydrolysis. Reactions were carried out in sodium phosphate buffer pH 7.0 at room temperature, as described in Section 3.9.

The use of soluble enzymes on an industrial scale is often costly and economically unviable, due to their disposal after use and poor stability under industrial conditions. Allowing biocatalyst reuse is one of the main advantages of immobilized enzymes both in batch and continuous processes, nevertheless, it was recently demonstrated that enzyme release from the hydrophobic supports can occur in the presence high concentration of free fatty acids both in aqueous and organic media, and it should be previously considered in the selection of an industrial lipase biocatalyst [17]. In order to evaluate the reusability, Oct and Phe derivatives of *Penicillium* sp. (CBMAI 1583) lipase were submitted to successive cycles of *p*-nitrophenyl palmitate (*p*NPP) hydrolysis (Figure 3).

Oct derivative showed a marked and constant activity decrease after each reaction cycle, retaining more than 50% of activity up to four cycles. On the other hand, the Phe derivative was fully stable up to the fourth cycle followed by a constant activity decrease. The activity loss may be due to enzyme release from the supports. The multiple and continuous operational steps and also the Triton X-100 used to prepare *p*NPP substrate must have collaborated for the enzyme desorption. Diverse results are generally observed when evaluating different lipase derivatives, e.g., Fadiloglu et al. [33] observed only 11% residual activity after three cycles with *Candida rugosa* lipase immobilized on Celite, while Cabrera-Padilla et al. [34] found that lipase from this microorganism immobilized on poly(3-hydroxybutyrate-co-ydroxyvalerate) can be reused up to 12 cycles, maintaining 50% of activity.

## 3. Materials and Methods

### 3.1. Materials

The supports octyl, butyl, and phenyl Sepharose, and cyanogen bromide activated (CNBr) 4BCL (4% crosslinking) agarose were purchased from GE Healthcare (Chicago, IL, USA) Toyopearl butyl 650 M was from Tosoh Bioscience (Tokyo, Japan), Lewatit VP OC 1600 was from Lanxess (Leverkussen, Germany), and Octadecyl Sepabeads was from Resindion (Binasco, Italy). Bovine serum albumin (BSA), bicinchoninic acid (BCA), *p*-nitrophenyl palmitate, sodium tetraborate, glycerol, ethanol, dimethyl sulfoxide, acetonitrile, cyclohexane, tert-amyl alcohol, Triton X-100, docosahexaenoic acid, and eicosapentaenoic acid were obtained from Sigma-Aldrich (St. Louis, MO, USA). Sardine oil (18% EPA and 12% DHA) was obtained from BTSA, Biotecnologías Aplicadas, S.L. (Madrid, Spain).

### 3.2. Strain Maintenance and Lipase Production

*Penicillium* sp. section *Gracilenta* (CBMAI 1583) was isolated from Atlantic Rainforest soil [20] and it is stored in the Brazilian Collection of Environmental and Industrial Microorganisms—CBMAI/CPQBA—UNICAMP, Paulínia, São Paulo, Brazil. Cultures were routinely maintained on oat-agar slants and stored at 4 °C. Conidia from five-day-old cultures were suspended in sterile distilled water to compose a 5 × 10^7^ conidia/mL suspension. For lipase production, one milliliter of this suspension was inoculated into 125 mL Erlenmeyer flasks containing 25 mL of culture medium: bacto peptone 5.0 g/L, yeast extract 1.0 g/L; NaNO_3_ 0.5 g/L; KCl 0.5 g/L; MgSO_4_·7H_2_O 0.5 g/L, KH_2_PO_4_ 2.0 g/L, and olive oil 5.0 g/L, pH 5.5. Cultivation was performed for three days, 160 rpm at 28 °C. Cultures were vacuum filtrated, lyophilized, and the resulting powder was used as lipase source.

### 3.3. Enzyme Activity and Protein Determination Assays

Enzyme activity was assayed by *p*NPP hydrolysis, which was performed under 500 rpm magnetic stirring for 2 min under controlled temperature, accompanying the released *p*-nitrophenolate (*p*NP) at 348 nm. The substrate was prepared by dissolving 3.8 mg of *p*NPP in 0.5 mL of DMSO and then diluting it to 0.5 mM with 25 mM sodium phosphate buffer pH 7.0 containing 0.5% (*w*/*v*) Triton X-100. To initialize the reaction, 0.1 mL of lipase solution or derivative suspension was added to 1.9 mL of reaction medium. One unit of enzyme activity was defined as the amount of enzyme necessary to release 1 μmol of *p*NP (ε = 5150) per minute under the assay conditions. Specific activity corresponded to the activity per gram of derivative.

Protein was determined with the Pierce BCA Protein Assay Kit, according to the manufacturer Thermo Fisher Scientific (Rockford, IL, USA), using BSA as standard.

### 3.4. Supports Preparation

The supports But, Phe, Oct, Toyo, Lew, and Sep were washed with distilled water and vacuum filtrated prior to use. CNBr was 1:35 (*w*/*v*) suspended in 0.1 M HCl pH 2.0 solution for 45 min. The swollen support was then washed with the same acid solution in order to remove additives and filtered under vacuum, as described by the manufacturer.

### 3.5. Enzyme Immobilization

For all immobilization protocols, 10 mg of powdered crude lipase were diluted in 1 mL of 5 mM sodium phosphate buffer pH 7.0, resulting in a 3.5 mg prot/mL lipase solution.

Adsorption on hydrophobic But, Phe, Oct, Toyo, Lew, and Sep supports was performed by adding 9 mL of lipase solution to 1.0 g of support. The mixture was kept at room temperature under mild agitation. After immobilization, the derivatives were vacuum filtrated, washed with 5 mM sodium phosphate buffer pH 7.0 and stored at 4 °C. Covalent immobilization on CNBr support was carried out with 4 mL of lipase solution added to one gram of support. The suspension was stirred for 15 min at 4 °C and vacuum filtrated.

Immobilization-course was followed by measuring lipase activity both on supernatant and suspension. A sample of soluble enzyme incubated under the same experimental conditions was the external control. Immobilization yield (*Y*) and recovered activity (*EA*) were calculated according to Equations (1) and (2), respectively:

(1) $$Y\;\left(\%\right)=\frac{A-B}{A}\;\times \;100$$

(2) $$EA\;\left(\%\right)=\frac{C}{A\;\times \;Y}\;\times \;100$$

in which *A* is the activity of the solution offered for immobilization, *B* is the activity in the supernatant at the end of immobilization, and *C* is the activity of the immobilized derivative. Experiments were performed in duplicates and standard error never exceeded 5%.

Enzyme desorption from the hydrophobic supports was evaluated by suspending one gram of the hydrophobic derivatives in 10 mL of 5 mM sodium phosphate buffer pH 7.0 at 25 °C followed by progressive addition of Triton X-100 from 0.1 to 10.0% (*w*/*v*). The immobilized enzymes were incubated under gentle stirring for 30 min before measuring the enzyme activity in the supernatant. A reference with the soluble enzyme under the same conditions was used to determine the effect of the detergent on enzyme activity.

### 3.6. Derivative Characterization

#### 3.6.1. Thermal Stability

Enzyme derivatives were 1:10 (*w*/*v*) suspended in 5 mM sodium phosphate buffer pH 7.0 and incubated at different temperatures. Samples were periodically withdrawn; lipase activity was measured and expressed in relation to the initial activity. First order deactivation rate constant (*K_d_*) and half-life (*t*_1/2_) were calculated according to Equations (3) and (4), respectively:

*ln A_i_ = ln A*_0_*– K_d_* × *t* (3)

(4) $$t_{1/2}=\frac{ln2}{K_{d}}$$

in which *A_i_* is the derivative specific lipase activity (U/g support) at time *t* (min) and *A*_0_ is lipase activity at time zero. The stabilization factor (SF) was calculated as the ratio between a derivative half-life and CNBr derivative half-life in a given temperature.

#### 3.6.2. Stability in Different pH

The derivatives were 1:10 (*w*/*v*) suspended in the following buffer systems: 0.5 M glycine-HCl pH 2.0 and 2.5, McIlvaine pH 3.0–8.0, 0.5 M Tris-HCl pH 8.5 and 9.0, and 0.5 M glycine-NaOH pH 9.5 and 10.0. The activity was measured after 24 h and expressed in relation to the initial activity.

#### 3.6.3. Stability in Different Organic Media

The octyl agarose derivative was 1:10 (*w*/*v*) suspended in organic media containing glycerol, ethanol, DMSO, acetonitrile, cyclohexane, and tert-amyl alcohol. Each substance was previously prepared at 50% (*v*/*v*) in 5 mM phosphate buffer pH 7.0. After incubation for 2 h at 25 °C, the activity was measured and expressed in relation to the initial activity.

### 3.7. Fish Oil Hydrolysis

Fish oil hydrolysis was performed with the soluble enzyme and with But, Phe, Oct, and CNBr derivatives in organic/aqueous biphasic system with cyclohexane, as proposed by Fernández-Lorente et al. [13]. The procedure was as follows: 2.25 mL cyclohexane, 2.5 mL McIlvaine buffer pH 5.0 and 0.25 mL fish oil were placed in a reactor and pre-incubated at 37 °C for 30 min; the reaction was then initialized by adding 0.3 g of derivative and stirred at 150 rpm for 24 h. The concentration of free fatty acids in the organic phase was determined by RP-HPLC (Spectra Physic SP 100 (Thermo Fisher Scientific, Waltham, MA, USA)) coupled with a UV detector SpectraPhysic SP 8450 (Thermo Fisher) using a reversed-phase column (Ultrabase C18, 4.6 mm i.d. × 150 mm, 5 μm particle, SFCC-Shandon, Eragny, France). Products were eluted with acetonitrile/water/acetic acid (70:30:0.1, *v*/*v*/*v*) pH 3.0 at 1.0 mL/min flow rate. Absorbance was read at 215 nm. Retention times (RT) for PUFA were 17–18 and 22–23 min for EPA and DHA, respectively. Produced PUFA were compared to their corresponding pure commercial standards and yields were calculated from the peak areas.

### 3.8. Fish Oil Ethanolysis

Fish oil ethanolysis was performed with Toyo, Lew, and Sep derivatives with cyclohexane, tert-amyl alcohol, as proposed by Moreno-Pérez et al. [31]. To initialize enzymatic synthesis of Omega-3 fatty acids ethyl esters, 0.3 g of the freeze-dried derivatives were added to the substrate solution. In reactions with solvents, substrate solution was composed of 0.59 mL sardine oil, 0.3 mL ethanol, 4.11 mL cyclohexane or tert-amyl alcohol and 0.2 g molecular sieves, at a 1:10 molar ratio between ethanol and fish oil (125 mM final concentration). In absence of cyclohexane and tert-amyl alcohol, 1.77 mL fish oil, 1.17 mL ethanol, and 0.2 g molecular sieves composed the reaction medium. The molar ratio was maintained and the oil final concentration was 701 mM. The reaction was carried out in an anhydrous system under mild stirring for 24 h at 45 °C. Reactants and products were analyzed by RP-HPLC (SpectraPhysic SP 100 coupled with a UV detector SpectraPhysic SP 8450) using a reversed-phase column (Ultrabase C18, 4.6 mm i.d. × 150 mm, 5 μm particle). Products were eluted with acetonitrile/water/acetic acid (80:20:0.1, *v*/*v*/*v*) pH 3.0 at 1.0 mL/min flow rate. Absorbance was read at 215 nm. Synthetic yields were calculated from pure peak areas corresponding to EE-EPA (RT = 24 min) and EE-DHA (RT = 28 min).

### 3.9. Derivative Reuse

Derivative reuse was performed in batch assays by incubating 0.1 g of phenyl and octyl Sepharose derivatives with one milliliter of *p*NPP solution (prepared as previously described) at room temperature. After 1 min reaction, the suspension was centrifuged (1 min, 5000× *g*, 4 °C) and the supernatant was transferred to tubes containing 1 mL of a saturated sodium tetraborate solution. The released *p*NP was measured at 405 nm (ε = 1.8 × 10^4^ M^−1^·cm^−1^). After each cycle, the derivatives were washed with 25 mM sodium phosphate buffer pH 7.0, filtrated and added to a new hydrolysis cycle with new substrate solution. The activity after each cycle was expressed in relation to the activity after the first cycle.

## 4. Conclusions

Adsorption on hydrophobic supports was a successful strategy for immobilization and stabilization of *Penicillium* sp. section *Gracilenta* (CBMAI 1583) lipase, since the hydrophobic derivatives are more stable to pH and temperature than the CNBr derivative, a model for enzyme behavior in the absence of intermolecular interactions. Besides, immobilization improved stability at high concentrations of glycerol, DMSO, and cyclohexane, an interesting characteristic for some industrial applications. The nature of the solvent is more decisive for the octyl derivative stability than the solvent hydrophobic properties. Immobilization on Sepabeads provides enzyme stabilization in a wide pH range. Besides, a 40-fold thermostabilization is achieved by the octyl Sepharose and Toyopearl butyl derivatives at 60 °C. For fish oil hydrolysis, phenyl Sepharose derivative is more active than the free enzyme, presenting higher specificity (ratio between EPA and DHA). The phenyl Sepharose is the most active derivative, with 1.2-fold higher activity than with the free enzyme and also shows higher specificity. When applied to fish oil ethanolysis, the Lewatit derivative shows higher activity in medium containing tert-amyl alcohol, presenting a 53-fold higher ethyl ester production. This study shows that the properties of the lipase from *Penicillium* sp. section *Gracilenta* (CBMAI 1583) can be modulated by directed immobilization—i.e., immobilization on different supports changes the catalytic properties, determining different selectivity consequently improving the biocatalyst properties.

## Figures and Tables

**Table 1:** Immobilization of *Penicillium* sp. (CBMAI 1583) lipase on hydrophobic supports.

Derivative	Yield (%)	Expressed Activity (%)	Specific Activity (U/mg Support)
Butyl Sepharose (But)	90.1	78.9	5.1
Phenyl Sepharose (Phe)	77.3	87.9	4.7
Octyl Sepharose (Oct)	71.4	144.9	20.9
Toyopearl butyl 650 M (Toyo)	90.3	73.6	3.7
Lewatit VPOC 1600 (Lew)	76.8	54.2	3.6
Octadecyl Sepabeads (Sep)	82.1	81.5	3.5

Lipase activity was measured with *p*-nitrophenyl palmitate (*p*NPP) and the activity of soluble lipase offered for immobilization was regarded as 100%. Immobilization parameters are described in Section 3.5.

**Table 2:** Thermal parameters of *Penicillium* sp. (CBMAI 1583) lipase derivatives.

Derivative	Parameter	Temperature (°C)		Stabilization ^c^
		50	60	
But	K_d_^a^ (h^−1^)	0.04	NM ^d^	-
	*t*_1/2_^b^ (h)	18.05		
Phe	K_d_^a^ (h^−1^)	0.10	NM ^d^	-
	*t*_1/2_^b^ (h)	6.05		
Oct	K_d_^a^ (h^−1^)	0.07	0.40	30.78
	*t*_1/2_^b^ (h)	9.74	1.72	
Toyo	K_d_^a^ (h^−1^)	0.02	0.27	46.25
	*t*_1/2_^b^ (h)	25.11	2.59	
Lew	K_d_^a^ (h^−1^)	0.53	NM ^d^	-
	*t*_1/2_^b^ (h)	1.30		
Sep	K_d_^a^ (h^−1^)	0.89	NM ^d^	-
	*t*_1/2_^b^ (h)	0.78		
CNBr	K_d_^a^ (h^−1^)	NM ^d^	12.36	-
	*t*_1/2_^b^ (h)		0.06	

^a^ K_d_—Deactivation constant; ^b^ *t*_1/2_—derivative half-life; ^c^ at 60 °C; ^d^ NM—not measured.

**Table 3:** Stability of the *Penicillium* sp. (CBMAI 1583) lipase octyl derivative (Oct) in organic media.

Solvent	Log *P*	Residual Activity (%)
Water	-	100
Glycerol	−1.67	93.11
DMSO	−1.35	41.90
Acetonitrile	−0.34	0.00
Ethanol	−0.30	0.00
Tert-amyl alcohol	0.89	0.00
Cyclohexane	3.44	48.79

Octyl derivative was incubated in 50% (*v*/*v*) solvent/ sodium phosphate buffer 5 mM pH 7, at 25 °C, for 2 h, under non-reactive conditions. Log *P*: logarithm of the partition coefficient of a particular solvent between *n*-octanol and water. DMSO: dimethyl sulfoxide.

**Table 4:** Sardine oil hydrolysis by *Penicillium* sp. (CBMAI 1583) lipase derivatives.

Derivative	Initial Activity ^a^	Selectivity ^b^
Free enzyme	0.078	4.78
But	0.032	3.47
Phe	0.093	5.68
Oct	0.073	2.27
CNBr	0.055	11.60

Reaction was carried out in an aqueous/organic biphasic system, with McIlvaine buffer pH 5.0/cyclohexane, at 45 °C, 150 rpm, and 24 h. ^a^ Initial activity is expressed as μmol of hydrolyzed polyunsaturated fatty acids (PUFA is the sum of eicosapentaenoic acid (EPA) and docosahexaenoic acid (DHA)) per minute per gram of immobilized enzyme. ^b^ Selectivity is expressed as the ratio between % of hydrolyzed EPA and % of hydrolyzed DHA.

**Table 5:** Sardine oil ethanolysis by *Penicillium* sp. (CBMAI 1583) lipase derivatives.

Solvent ^a^	Derivative	Initial Activity ^b^	Selectivity ^c^
-	Sep	0.130	2.82
	Lew	0.172	2.31
	Toyo	0.074	1.44
Cyclohexane	Sep	0.012	1.97
	Lew	0.189	1.93
	Toyo	-	-
Tert-amyl alcohol	Sep	0.020	2.20
	Lew	0.988	2.07
	Toyo	0.017	-

Reactions were carried out in anhydrous system under mild stirring for 24 h at 45 °C. ^a^ Other than ethanol. ^b^ Initial activity is expressed as μmol of ethyl esters of PUFA (EPA DHA) synthesized per hour per mg of immobilized protein. ^c^ Selectivity is expressed as the ratio between % of synthesized EPA and % of synthesized DHA.

## Supplementary Materials

Supplementary materials are available online.
