# Supplementary material for: Immobilization of Lipase from Penicillium sp. Section Gracilenta (CBMAI 1583) on Different Hydrophobic Supports: Modulation of Functional Properties
Source: Molecules. 2017 Feb 22;22(2):339. doi: 10.3390/molecules22020339 (PMC6155730; doi:10.3390/molecules22020339)
Supplement: Supplementary file 1 [file molecules-22-00339-s001.pdf]

Supplementary Materials: Immobilization of Lipase from *Penicillium* sp. Section *Gracilenta* (CBMAI 1583) on Different Hydrophobic Supports: Modulation of Functional Properties

Daniela F. M. Turati, Wilson G. Morais Júnior, César R. F. Terrasan, Sonia Moreno-Perez, Benevides C. Pessela, Gloria Fernandez-Lorente, Jose M. Guisan, and Eleonora C. Carmona

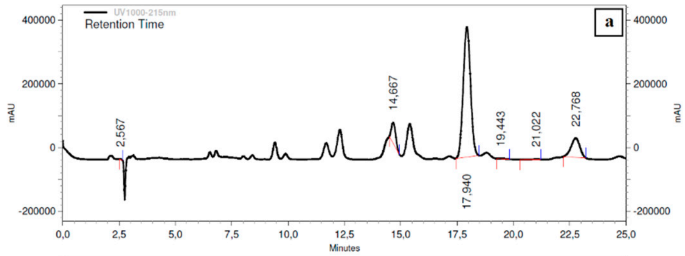

Retention Time	Area	Area Percent
2,567	12061	0,102
14,667	891440	7,564
17,940	9218890	78,227
19,443	20932	0,178
21,022	21400	0,182
22,768	1620090	13,747

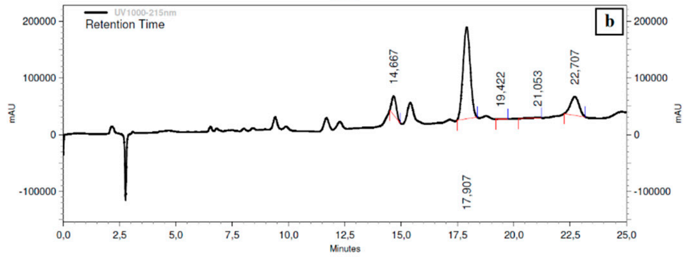

Retention Time	Area	Area Percent
14,667	471861	9,476
17,907	3606092	72,418
19,422	5038	0,101
21,053	22495	0,452
22,707	874038	17,553

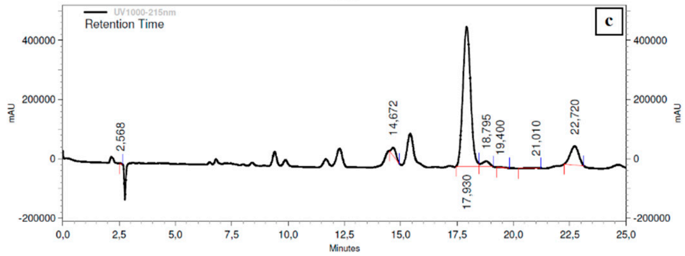

Retention Time	Area	Area Percent
2,568	7072	0,052
14,672	393297	2,869
17,930	11189338	81,637
18,795	416127	3,036
19,400	19922	0,145
21,010	25385	0,185
22,720	1655130	12,076

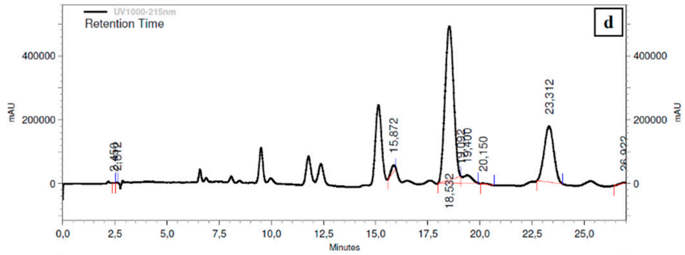

Retention Time	Area	Area Percent
2,450	838	0,004
2,612	2977	0,014
15,872	287265	1,318
18,532	15063183	69,099
19,092	0	0,000
19,400	785444	3,603
20,150	38953	0,179
23,312	5578509	25,590
26,922	42217	0,194

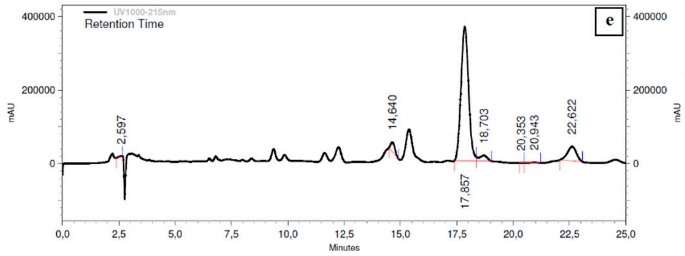

Retention Time	Area	Area Percent
2,597	10988	0,106
14,640	351308	3,392
17,857	8528975	82,356
18,703	363206	3,507
20,353	268	0,003
20,943	20205	0,195
22,622	1081244	10,441

Figure S1. Elution profiles on RP-HPLC of sardine oil hydrolysis by the free and immobilized lipase from *Penicillium* sp. (CBMAI 1583). Hydrolysis reactions and analysis were carried out as described in Materials and Methods section by the free enzyme (**a**); butyl derivative (**b**); phenyl derivative (**c**); octyl derivative (**d**) and CNBr derivative (**e**). Retention times (RT) for polyunsaturated fatty acids (PUFA) were 17–18 and 22–23 min for eicosapentaenoic acid (EPA) and docosahexaenoic acid (DHA), respectively. Units for labels in graphic axes and in tables: *y*-axis: mAU (mili arbitrary units); Retention time (min), Area (arbitrary units), and Area Percent (%).
